# Increased Protein *S*-Glutathionylation in Leber’s Hereditary Optic Neuropathy (LHON)

**DOI:** 10.3390/ijms21083027

**Published:** 2020-04-24

**Authors:** Lei Zhou, James Chun Yip Chan, Stephanie Chupin, Naïg Gueguen, Valérie Desquiret-Dumas, Siew Kwan Koh, Jianguo Li, Yan Gao, Lu Deng, Chandra Verma, Roger W Beuerman, Eric Chun Yong Chan, Dan Milea, Pascal Reynier

**Affiliations:** 1 Ocular Proteomics, Singapore Eye Research Institute, Singapore 169856, Singapore; koh.siew.kwan@seri.com.sg (S.K.K.); lijg@bii.a-star.edu.sg (J.L.); gao.yan@seri.com.sg (Y.G.); rwbeuerman@gmail.com (R.W.B.); 2 Department of Ophthalmology, Yong Loo Lin School of Medicine, National University of Singapore, Singapore 119228, Singapore; 3 Ophthalmology and Visual Sciences Academic Clinical Research Program, Duke-NUS Medical School, National University of Singapore, Singapore 169857, Singapore; 4 Department of Pharmacy, National University of Singapore, 18 Science Drive 4, Singapore 117543, Singapore; james_chan@sris.a-star.edu.sg (J.C.Y.C.); eric.chan@nus.edu.sg (E.C.Y.C.); 5 Département de Biochimie et Génétique, Centre Hospitalier Universitaire, 49933 Angers, France; Stephanie.Chupin@chu-angers.fr (S.C.); NaGueguen@chu-angers.fr (N.G.); VaDesquiret@chu-angers.fr (V.D.-D.); 6 Unité Mixte de Recherche (UMR) MITOVASC, Centre National de la Recherche Scientifique (CNRS) 6015, Institut National de la Santé et de la Recherche Médicale (INSERM) U1083, Université d’Angers, 49933 Angers, France; 7 Atomistic Simulations and Design in Biology, Bioinformatics Institute, 30 Biopolis Street, #07–01 Matrix, Singapore 138671, Singapore; chandra@bii.a-star.edu.sg; 8 Department of Statistics and Applied Probability, Faculty of Science, National University of Singapore, Singapore 117546, Singapore; e0046901@u.nus.edu; 9 Department of Biological Sciences, National University of Singapore, 16 Science Drive 4, Singapore 117558, Singpaore; 10 School of Biological Sciences, Nanyang Technological University, 60 Nanyang Drive, Singapore 637551, Singapore; 11 Singapore Institute for Clinical Sciences, Brenner Centre for Molecular Medicine, 30 Medical Drive, Singapore 117609, Singapore; 12 Département d’Ophtalmologie, Centre Hospitalier Universitaire, 49933 Angers, France; 13 Neuro-Ophthalmology Department, Singapore National Eye Centre, Singapore 168751, Singpaore

**Keywords:** Leber’s Hereditary Optic Neuropathy, LHON, *S*-glutathionylation, proteomics, mitochondrial Complex I

## Abstract

Leber’s hereditary optic neuropathy (LHON, MIM#535000) is the most common form of inherited optic neuropathies and mitochondrial DNA-related diseases. The pathogenicity of mutations in genes encoding components of mitochondrial Complex I is well established, but the underlying pathomechanisms of the disease are still unclear. Hypothesizing that oxidative stress related to Complex I deficiency may increase protein *S*-glutathionylation, we investigated the proteome-wide *S*-glutathionylation profiles in LHON (*n =* 11) and control (*n =* 7) fibroblasts, using the GluICAT platform that we recently developed. Glutathionylation was also studied in healthy fibroblasts (*n =* 6) after experimental Complex I inhibition. The significantly increased reactive oxygen species (ROS) production in the LHON group by Complex I was shown experimentally. Among the 540 proteins which were globally identified as glutathionylated, 79 showed a significantly increased glutathionylation (*p* < 0.05) in LHON and 94 in Complex I-inhibited fibroblasts. Approximately 42% (33/79) of the altered proteins were shared by the two groups, suggesting that Complex I deficiency was the main cause of increased glutathionylation. Among the 79 affected proteins in LHON fibroblasts, 23% (18/79) were involved in energetic metabolism, 31% (24/79) exhibited catalytic activity, 73% (58/79) showed various non-mitochondrial localizations, and 38% (30/79) affected the cell protein quality control. Integrated proteo-metabolomic analysis using our previous metabolomic study of LHON fibroblasts also revealed similar alterations of protein metabolism and, in particular, of aminoacyl-tRNA synthetases. *S*-glutathionylation is mainly known to be responsible for protein loss of function, and molecular dynamics simulations and 3D structure predictions confirmed such deleterious impacts on adenine nucleotide translocator 2 (ANT2), by weakening its affinity to ATP/ADP. Our study reveals a broad impact throughout the cell of Complex I-related LHON pathogenesis, involving a generalized protein stress response, and provides a therapeutic rationale for targeting *S*-glutathionylation by antioxidative strategies.

## 1. Introduction

Leber’s hereditary optic neuropathy (LHON, MIM#535000) is the most common mitochondrial DNA-related disorder and the most common form of inherited optic neuropathies, with a prevalence greater than 3/100,000 [1]. LHON is due to the degeneration of retinal ganglion cells transmitting the visual signal from the retina to the visual cortex. It is clinically characterized by subacute and painless loss of vision occurring in one eye followed by the involvement of the other eye within weeks or months [2,3,4]. The visual prognosis is usually poor, leading to legal blindness. Three main mitochondrial DNA (mtDNA) pathogenic variants with variable penetrance account for about 90–95% of LHON cases. They are located in NADH dehydrogenase (ND) genes encoding Complex I subunits of the mitochondrial respiratory chain, at nucleotide positions m.11778G>A (*MT-ND4*), for the most common mutation with the worst visual prognosis, and at m.3460G>A (*MT-ND1*) and m.14484T>C (*MT-ND6*). Previous studies have shown respiratory chain Complex I deficiency [5], oxidative stress [6], increased susceptibility to cell death [7], and defect of compensatory mitochondrial biogenesis [8].

It is believed that the mitochondrial Complex I is the key source of reactive oxygen species (ROS) and the major contributor to cellular oxidative stress [9]. Oxidative stress has been associated with increased *S*-glutathionylation [10,11], but little is known about its pathogenic implications in LHON. The whole-cell consequences of redox imbalance generated by energetic deficiency in mitochondrial disorders, and more specifically by Complex I deficiency in LHON, have never been investigated at an omics scale to our knowledge. The exploration of proteome-wide glutathionylation, measuring the redox-sensitive proteome, provides an opportunity to gain a global idea of the cellular functions that the mitochondrial deficit and redox imbalance can perturb.

Protein *S*-glutathionylation is a reversible post-translational modification in which protein cysteine residues form a disulfide bond with the cysteine of tripeptide glutathione (GSH) [12]. Protein glutathionylation is mainly enzymatically or spontaneously driven through direct oxidation or by thiol–disulfide exchange. Oxidative stress increases glutathione disulfide (GSSG) production, leading to increased *S*-glutathionylation of redox-sensitive proteins through thiol–disulfide exchange, as shown in vitro or in many animal models of diseases [13,14]. Protein glutathionylation is mainly known to lead to the loss of function of these proteins [13].

*S*-glutathionylation has various mitochondrial targets [15], including the Krebs cycle, pyruvate dehydrogenase, Complex I, II and V of the respiratory chain, transmembrane carriers (ANT, UCP1–3, VDAC), and various proteins involved in apoptosis (AIF, cyclophilin-D). The aim of this study was to evaluate the *S*-glutathionylation profile in LHON, as a consequence of Complex I deficiency and oxidative stress.

## 2. Results

### 2.1. Population

The baseline characteristics of LHON and control fibroblasts are described in Table 1. The m.11778G>A, m.14484T>C, and m.3460G>A mtDNA variants were carried by eight, two, and one LHON fibroblasts, respectively. The mean age of patients at the time of skin biopsies was 32 years (range: 19–58 years, median: 26 years) for LHON patients and 30 years for controls (range: 8–56 years, median: 30 years), with 8/11 and 3/7 males in the two groups, respectively.

### 2.2. Complex I Impairment in LHON and Treated Fibroblasts

**Figure 1:**
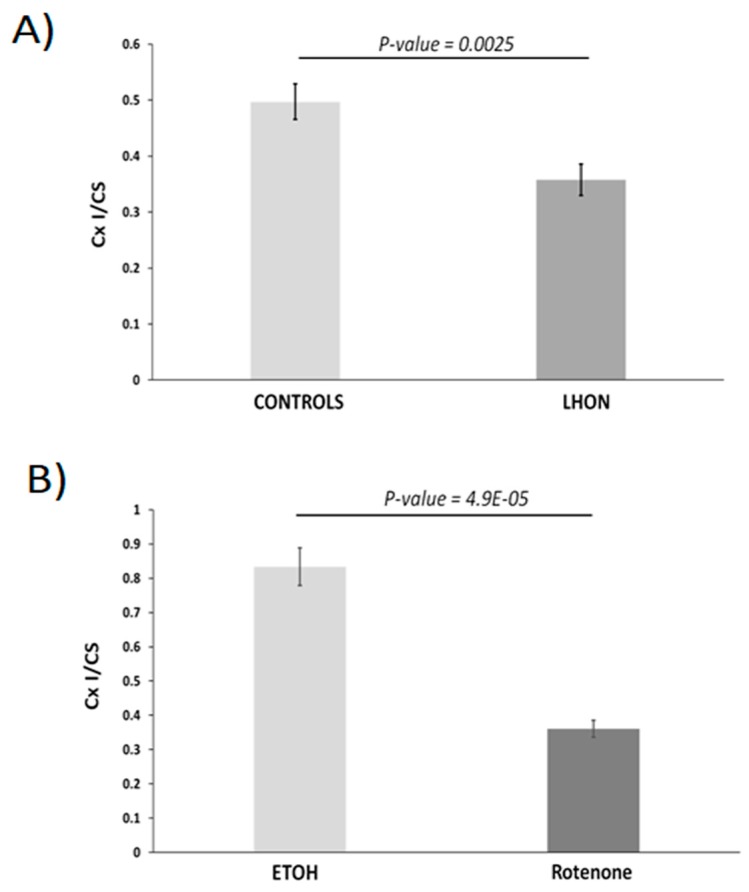
Enzymatic activity of Complex I (Cx I) in LHON fibroblasts and in control fibroblasts inhibited by rotenone. (**A**) Activity in fibroblasts from LHON (*n* = 11 in duplicate) and controls (*n* = 7 in duplicate). Compared to controls, the average Complex I enzymatic activity in LHON fibroblasts was reduced by 28% (*p* = 0.0025). (**B**) Activity in control fibroblasts treated with the vehicle (ethanol, *n* = 6) or treated with Complex I inhibitor (rotenone 1 μM, *n* = 6). Complex I inhibition was 57% (*p* = 4.9 × 10^5^) in treated cells compared to controls. Results were normalized with respect to citrate synthase (CS) activity (Cx I/CS). Statistical significance: * *p* < 0.05 and ** *p* < 0.01.

Compared to controls, the average Complex I enzymatic activity in LHON fibroblasts was reduced by 28% (*p* = 0.0025) (Figure 1A). Complex I inhibition was 57% (*p* = 4.9 × 10^−5^) in treated cells compared to control fibroblasts (Figure 1B).

### 2.3. Complex I Deficiency Induces ROS Overproduction.

**Figure 2:**
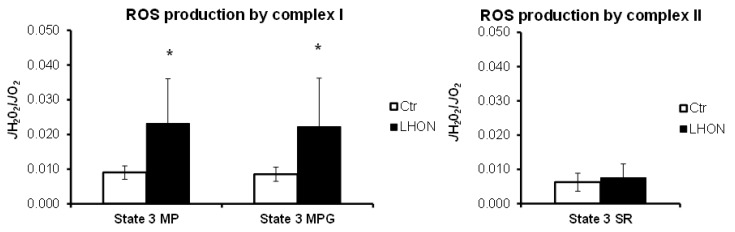
LHON and control fibroblast ROS production. ROS production was measured simultaneously with oxygen consumption using the O2k-Fluorometer equipped with two-channel fluorescence optical setup to monitor oxygen level and fluorescence. State 3 MP: maximal phosphorylating respiration with Complex I substrates malate (5 mM) and pyruvate (2.5 mM). State 3 MPG: maximal phosphorylating respiration with Complex I substrates malate (5 mM), pyruvate (2.5 mM), and glutamate (5 mM). State 3 SR: maximal phosphorylating respiration with Complex II substrate succinate (10 mM) and Complex I inhibited by rotenone (5 µM). Statistical significance *: *p* < 0.05.

O_2_ and H_2_O_2_ fluxes were determined in parallel on the same experiment for each sample, using either Complex I substrates, i.e., pyruvate and malate (PM) or pyruvate, malate, and glutamate (PMG), or Complex II substrate, i.e., succinate with rotenone (SR, rotenone being used to inhibit the reverse electron transfer to Complex I) (Figure 2). The relative H_2_O_2_/O_2_ flux ratios reflect the relative importance of H_2_O_2_ production according to the different oxidized substrates [16]. The ROS production by Complex I was significantly increased in the LHON group, using PM (+157%, *p* = 0.0047) or PMG (+161%, *p* = 0.012) substrates. As expected, the Complex II-linked ROS production did not show a significant difference between LHON and control groups (+21%, *p* = 0.62). These results demonstrate a specific increased ROS production due to Complex I dysfunction in LHON fibroblasts.

### 2.4. Phosphorylating Respiration and ATP Production in LHON Permeabilized Fibroblasts

**Figure 3:**
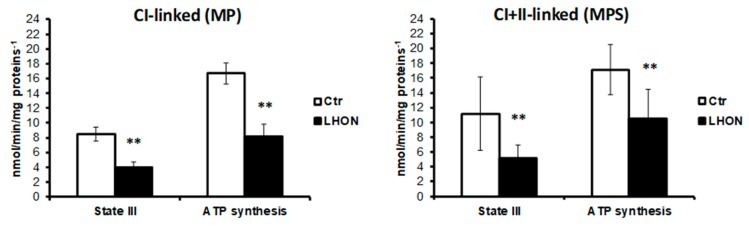
Maximal phosphorylating respiration rate (state III) and the corresponding mitochondrial ATP synthesis rate were determined in LHON (*n* = 7) and control (*n* = 7) fibroblasts. State III was started either by addition of 5 mM malate and 2.5 mM pyruvate (Complex I-linked respiration) or of 5 mM malate, 2.5 mM pyruvate, and 10 mM succinate (complexes I+II-linked respiration), and phosphorylating respiration was induced by the subsequent addition of 1.5 mM ADP. Statistical significance: ** *p* < 0.01.

We further analyzed the rate of maximal ATP synthesis with either Complex I (malate + pyruvate) or Complex I+II (malate, pyruvate and succinate) substrates. As Figure 3 shows, the ATP synthesis was significantly reduced in LHON fibroblasts, with substrate oxidation by Complex I (*p* = 0.0031) and by Complexes I and II (*p* = 0.0030).

### 2.5. Protein S-glutathionylation profile in LHON fibroblasts

**Figure 4:**
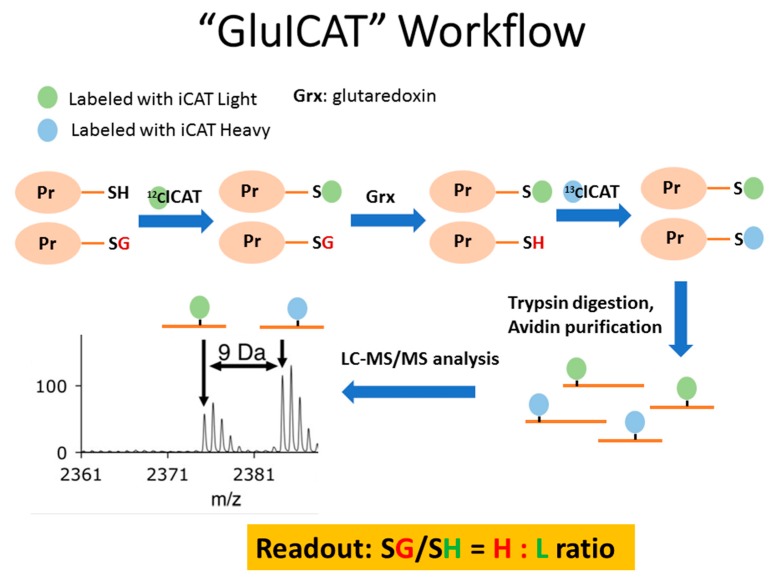
Workflow for quantitative analysis of proteome-wide glutathionylation using our previously developed strategy (GluICAT) [17]. Reduced thiols are labeled with light ICAT (^12^C). Glutathionylated thiols were specifically cleaved using glutaredoxin (Grx) and subsequently labeled with heavy ICAT (^13^C). After tryptic digestion and avidin enrichment, peptides were analyzed using LC-MS/MS. The extent of glutathionylation (SG/SH) of a peptide can be determined by the ratio of heavy-to-light ICATs (H:L).

The validation of the methodology for the quantitative analysis of proteome-wide *S*-glutathionylation was reported recently by our group [17]. The same proteomic workflow was utilized in this study (Figure 4, [17]).

A total of 2133 peptides (confidence > 95%, 2307 *S*-glutationylation sites) corresponding to 540 proteins (FDR < 1%) were found to be glutathionylated (Supplemental Table S1). Noticeably, GO analysis revealed pleiotropic functions (Supplemental Figure S1A) of these proteins, with 35.8% of them exhibiting catalytic activity (Supplemental Figure S1B).

**Figure 5:**
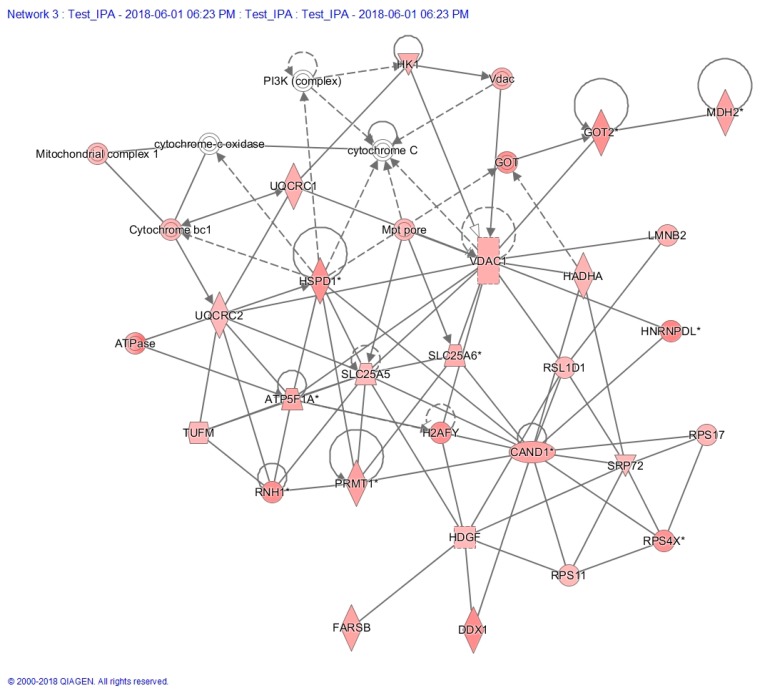
IPA pathway analysis shows one of the enriched networks (Network 3), which is related to energy production.

**Figure 6:**
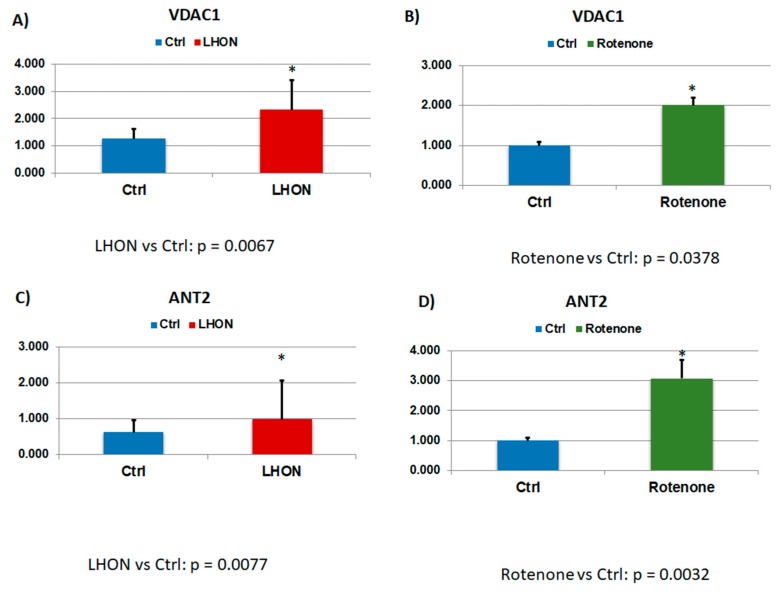
Increased level of glutathionylation in VDAC1 was observed in (**A**) LHON as compared with controls (*p* = 0.0067) and (**B**) Complex I inhibition by Rotenone as compared with controls (*p* = 0.0387). Increased level of glutathionylation in ANT2 was observed in (**C**) LHON as compared with controls (*p* = 0.0077) and (**D**) Complex I inhibition by Rotenone as compared with controls (*p* = 0.0032). Y-axis values refer to ratios of glutathionylated vs. un-glutathionylated. Statistical significance: * *p* < 0.05.

Based on the cutoff of [H:L disease/H:L control] ratio greater than 1.5-fold, increased levels of glutathionylation were found in 438 peptides corresponding to 254 proteins (79 proteins with *p* < 0.05, Supplemental Table S2). GO analysis again showed pleiotropic functions (Supplemental Figure S2A) with 30.6% of these proteins exhibiting catalytic activity (Supplemental Figure S2B). IPA molecular network analysis identified 14 enriched networks (Supplemental Table S3). One of the top networks was related to energy production (network 3, score = 47; number of focus molecules = 26, Supplemental Table S3, Figure 5). Network 3 clearly showed (Figure 5) that the mitochondrial proteins (21/79, 27.0%) were enriched in this dataset, with increased glutathionylation of many mitochondrial proteins, including ATPase, SLC25A5 (ANT2), SLC25A6 (ANT3), VDAC1, and GOT2, etc. Overall, 23% (18/79) hyper-glutathionylated proteins were involved in energy metabolism. The glutathionylation levels of VDAC1 and ANT2 from LHON patients and healthy controls are presented in Figure 6A,C. The ratios of [H:L disease / H:L control] for VDAC1 and ANT2 were 1.85 (*p* = 0.0067, Figure 6A) and 1.59 (*p* = 0.0077, Figure 6C), respectively. Many other bioenergetic pathways were also affected, such as glycolysis and gluconeogenesis, carbohydrate metabolism, the TCA cycle, pyruvate and ubiquinone metabolisms, as well as metabolic pathways involving amino acids. Surprisingly, 38% (30/79) of glutathionylated proteins were involved in different stages of protein metabolism (protein synthesis, folding, trafficking, and degradation) (proteins tagged in green in Supplemental Table S2). Overall, 73% (58/79) proteins with increased glutathionylation had non-mitochondrial localization showing a broader cell impact of LHON.

### 2.6. Protein S-Glutathionylation Profile in Fibroblasts with Complex I Inhibition

**Figure 7:**
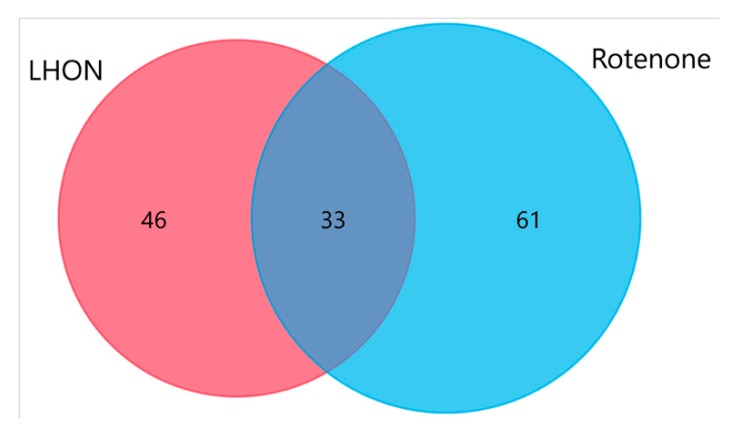
Venn diagram of elevated levels of glutathionylated proteins in LHON and Complex I-inhibited fibroblasts.

To confirm whether increased protein *S*-glutathionylation was directly related to Complex I deficiency, the protein *S*-glutathionylation profile of Complex I-inhibited fibroblasts (induced by treatment with rotenone) was compared with that of vehicle-treated fibroblasts. Similarly, using the cutoff of [H:L treated / H:L control] ratio exceeding 1.5-fold, increased levels of glutationylation were found in 94 proteins (Supplemental Table S4). There was a significant overlap between LHON and Complex I inhibited fibroblasts, with 42% (33/79) of the proteins belonging to the two groups (Figure 7). Similarly, with the LHON fibroblasts, the ratios of [H:L treated / H:L control] for VDAC1 and ANT2 were 1.85 (*p* = 0.0067, Figure 6B) and 1.59 (*p* = 0.0077, Figure 6D), respectively. Again, many glutathionylated proteins were involved in the bioenergetic, amino acid, and protein metabolisms. The majority of the hyper-glutathionylated proteins (71/94, 75.5%) had a non-mitochondrial origin, which shows the extra-mitochondrial impact of Complex I inhibition on glutathionylation.

### 2.7. Proteo-Metabolomic Mapping of S-Glutathionylation in LHON

**Figure 8:**
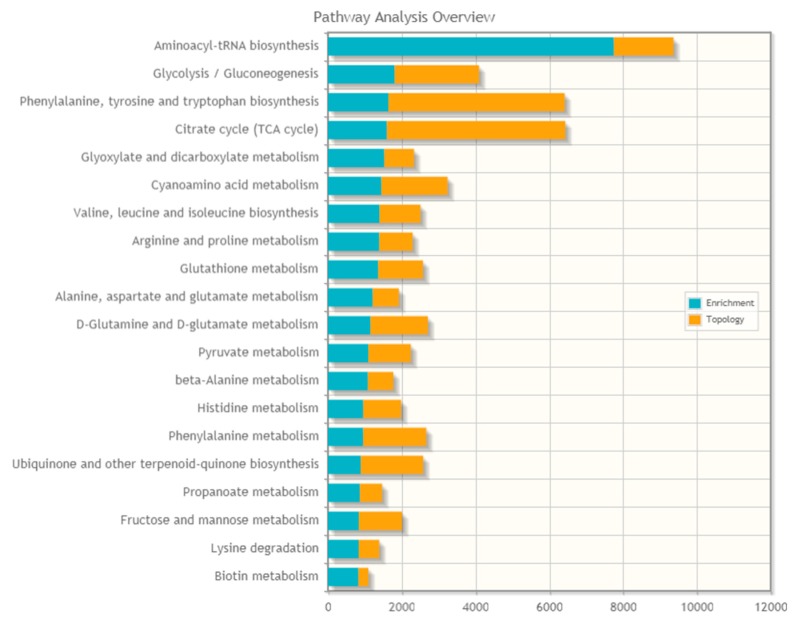
Proteo-metabolomic mapping of glutathionylated proteins in LHON.

We integrated the protein *S*-glutathionylation dataset with the metabolomics dataset from our previous study performed on LHON fibroblasts compared to controls that showed significant differences in the concentrations of the following metabolites: 10 phosphatidylcholines, 19 amino acids, two acylcarnitines, five sphingomyelins, and two biogenic amines [18]. Using the “Joint pathway analysis” module in “Metaboanalyst” [19], we found that the top five affected pathways were aminoacyl-tRNA biosynthesis, glycolysis/gluconeogenesis, phenylalanine/tyrosine/tryptophan biosynthesis, the TCA cycle, and glyoxylate/dicarboxylate metabolism (Figure 8).

### 2.8. Molecular Dynamic Simulations of Native and Glutathionylated ANT2

**Figure 9:**
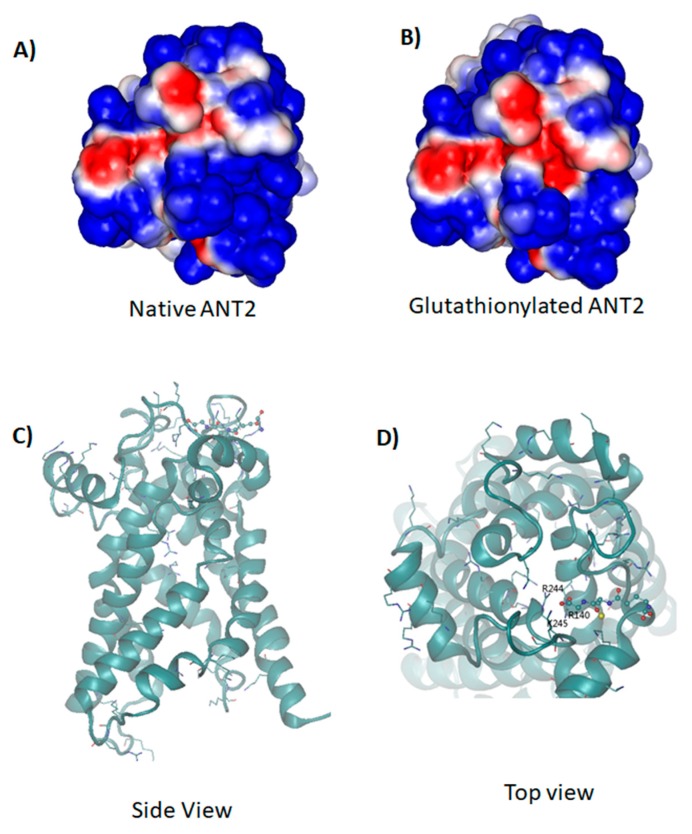
Molecular dynamic simulations of (**A**) native and (**B**) glutathionylated ANT2 (*p*.Cys160) show that glutathionylation results in more negative (blue indicates positive potential and red indicates negative potential) electrostatic surface potential at the entrance of the pore, which thus weakens the affinity of ATP/ADP to the ANT2 protein. The cartoon representation [Side view (**C**) and top view (**D**)] of the glutathionylated ANT2. The glutathionylated group is shown in CPK, and basic residues (e.g., Arg and Lys) are shown in bonds. The glutathionylated group can form hydrogen bonds to the adjacent basic residues. This may reduce the conformational flexibility of the ANT2 protein, which is required for the translocation of ADP/ATP across the pore. However, the predicted outcome needs further confirmation from experimental evidence.

The homology modelling of the structure of glutathionylated ANT2 revealed that the glutathionylation of Cys160 locates near the entrance of the ANT2 channel; therefore, glutathionylation may affect the dynamics and the exchange of ADP and ATP via the ANT2 channel. The glutathionyl group carries a net negative charge, thus glutathionylation modifies the electrostatic field along the channel, as can be seen from the electrostatic surface potential of the native and glutathionylated ANT2 molecules from MD simulations (Figure 9A,B). In native ANT2 protein, the surface is largely positive, which provide the electrostatic driving force for the binding of negatively charged ADP and ATP molecules. While in glutathionylated ANT2 molecules, due to the presence of the glutathionyl group, the surface potential is less positive, suggesting decreased affinity for ATP and ADP molecules. It has been shown that in the native ANT2, the translocation of ATP/ADP is largely mediated by the electrostatic interactions along the ANT2 channel [20]. The shift of the electrostatic potential towards a negative value affects the ATP/ADP permeability across the ANT2 channel. In addition, the COO^-^ groups in the glutathionyl group could further form hydrogen bonds with adjacent basic residues (Figure 9C,D), which may result in reduced conformational flexibility of the ANT2 protein. As the ANT2 protein needs to undergo a conformational change during ATP/ADP translocation, the reduction in the molecular flexibility will also affect its channel permeability.

## 3. Discussion

**Figure 10:**
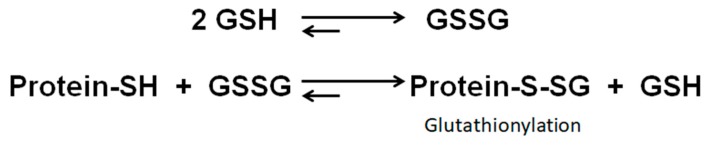
An imbalance between reduced glutathione (GSH) and oxidized glutathione disulfide (GSSG) can induce the formation of greater glutathionylation of proteins.

In this study, we have demonstrated that LHON is associated with a significant increase of the cell proteome glutathionylation, significantly affecting 79 proteins (*p* < 0.05). Thus, in LHON, the oxidative stress generated by Complex I deficiency increases ROS production [21], as shown in our experiments, should create an imbalance between reduced glutathione (GSH) and oxidized glutathione disulfide (GSSG) and subsequently induce increased levels of protein glutathionylation (Figure 10). In support of this hypothesis, experimental inhibition of Complex I in healthy fibroblasts by rotenone, which is known to induce ROS overproduction by Complex I, induced similar proteomic changes, suggesting that increased glutathionylation may be a direct consequence of Complex I deficiency. Interestingly, principal component analysis (PCA) suggested that m.11778G>A and m.3460G>A cluster together, and m.14484T>C clusters together with controls (figure not shown because there were only two cases for m.14484T>C and one case for m.3460G>A in this study).

Our study has also shown that among the proteins with increased glutathionylation levels in LHON, only a quarter (27%) were of mitochondrial origin and the remainder, 73%, had various extra-mitochondrial localizations, in the endoplasmic reticulum, Golgi apparatus and derived vesicles, lysosomes, nucleus, ribosomes, and cell membranes. Functionally, this large set of affected proteins has pleiotropic roles in metabolism, gene expression, cell signaling, and cell structures. Proteo-metabolomic mapping of *S*-glutathionylation in LHON fibroblasts showed that aminoacyl-tRNA, glycolysis/gluconeogenesis, phenylalanine/tyrosine/tryptophan biosynthesis, the TCA cycle, and glyoxylate/dicarboxylate metabolisms were the top five affected pathways in this condition, with glutathione metabolism itself also being disturbed (Figure 8). Indeed, three glutathione-related enzymes were hyper-glutathionylated, the microsomal glutathione S-transferase 1 (MGST1), located both in the endoplasmic reticulum and mitochondria and involved in electrophilic compound detoxication, glutathione S-transferase Pi 1 (GSTP1) which is also involved in electrophilic compound detoxication, and mitochondrial glutathione reductase (GSR) involved in the reduction of oxidized GSSG into GSH. The activities of these three antioxidant enzymes may be affected by their own increased glutathionylation, since glutathionylation is usually known to affect enzymatic activities [12,22].

Our results show that LHON is not restricted to its mitochondrial environment but has a much broader deleterious impact throughout the cell. We found that 40% of the hyper-glutathionylated proteins exhibited catalytic activity, and it is likely that most of these enzymes may be inhibited in LHON fibroblasts with broad deleterious consequences.

Interestingly, at least one third of the hyper-glutathionylated proteins are involved in the different steps of the protein metabolism. In particular, we noticed that the increased glutathionylation levels of a group of amino-tRNA ligases (also called aminoacyl-tRNA synthetases), including valine-tRNA ligase, arginine-tRNA ligase, phenylalanine-tRNA ligase, glutamine-tRNA ligase, aspartate-tRNA ligase, and glutamate/proline-tRNA ligase (Supplemental Table S2), featured LHON fibroblasts compared to controls. Most (17/19) mitochondrial aminoacyl-tRNA synthetases have been reported to cause neurodegeneration [23]; this feature could play an important pathogenic role in LHON [24].

Protein synthesis is one of the most energy consuming biological processes [25], and the alteration of protein metabolism could directly result from the bioenergetic defect and may lead to endoplasmic reticulum stress. Using a metabolomic approach for the same collection of LHON and control fibroblasts, we previously suggested an activation of the endoplasmic reticulum stress in this condition [8], a finding which was also suggested by transcriptomic [26] and proteomic studies [27]. Thus, all these omics studies reinforce the concept of possible generalized protein stress as a response to the disease.

To illustrate the consequence of protein *S*-glutathionylation in LHON, we further investigated how it may affect ANT2 (sometimes called ADT2), a mitochondrial protein carrier involved in ADP and ATP exchange across the mitochondrial inner membrane. ANT2 is highly positively charged due to the presence of large number of arginine and lysine residues. This creates a favorable electrostatic potential driving the binding of negatively charged ADP and ATP to ANT2. As suggested by molecular dynamic simulation, the negatively charged glutathionylated groups locate at the entrance of the pocket of ANT2 and reduce the positive charges which may weaken the affinity of ADP/ATP to the ANT2 protein. Furthermore, it is reported that during ATP/ADP translocation, the ANT2 protein undergoes a conformational change. The presence of additional anionic COO^-^ groups in glutathionylated ANT2 leads to the formation of hydrogen bonds with basic residues. This may reduce the conformational flexibility of the ANT2 protein and results in an enhanced free energy barrier for the conformational change.

Recent reports suggested that ANT, together with the voltage-dependent anion channel (VDAC), plays critical roles in apoptosis. ANT cooperates with Bax and the outer membrane VDAC to form a mitochondrial permeability transition pore (MPTP) involved in apoptosis [28]. Increased levels of glutathionylation of ANT2, ANT3, and VDAC1 were observed in LHON fibroblasts. The molecular dynamic simulation of VDAC1 and the impact of glutathionylation on the molecular structure of VDAC1 were analyzed in detail in our previous study [17]. Briefly, glutathionylated VDAC1 shifts the conformation of native VDAC to an open state causing by the hydrogen bonds between the glutathionyl group with the VDAC1 protein. This hyper-glutathionylation may impair ATP/ADP exchange across the mitochondrial membrane and may lead to a shift of MPTP to a more open state, explaining the increased susceptibility of LHON fibroblasts to cell death [29]. This result is reinforced by the increased glutathionylation in LHON fibroblasts of apoptosis-mitochondrial inducing factor 1 (AIFM1), a deficiency of which leads to severe mitochondrial dysfunction [30].

The stoichiometry (or occupancy) of *S*-glutathionylation on a specific cysteine residue is important for understanding the functional role of the modification. Previous work on mouse macrophages showed that the basal average oxidation level of protein thiols was ~12%, and the average percentage of basal *S*-glutathionylation relative to total oxidation was 32.0%, suggesting that *S*-glutathionylation is one major type of thiol modification [31]. Although the stoichiometry of *S*-glutathionylation of a protein is around 1–5%, collectively, increased *S*-glutathionylation of many proteins may cause significant effects on the function of the whole pathway, for example, many mitochondrial proteins are affected.

Currently there is no proven treatment for LHON but antioxidant treatments (such as coenzyme Q10, idebenone, and EPI-743) can reduce the oxidative stress induced by the disease [32]. Our results suggest that if the imbalance between glutathione GSH and GSSG could be restored by various antioxidant therapies, this may result in a reduction of protein glutathionylation levels and improved enzymatic activity, at a large scale.

The main limitation of this study is the small number of fibroblast cell lines from LHON and controls explored. However, the similarity of glutathionylation profile obtained in LHON and in experimental Complex I inhibition is in favor of a sharp alteration of the proteome. It will be interesting to see in future studies whether the glutathionylation LHON profile presented here will also be found in other forms of mitochondrial diseases or optic neuropathies.

In conclusion, we present the first proteome-wide glutathionylation study performed on a mitochondrial DNA-related disease. Our results suggest that LHON is associated with globally increased protein glutathionylation, throughout the cell, affecting bioenergetics pathways but also many non-mitochondrial proteins. As it has been shown through other omics approaches, our study points to a sharp impact of LHON on the whole cell protein metabolism, attesting to an integrated stress response due to Complex I defect. The deleterious impact of glutathionylation has been illustrated on ANT2 (glutathionylation weakens its affinity for ATP/ADP) and VDAC (glutathionylation shifts its conformation to an open state), using protein structure prediction tools. Our data provide the rationale for targeting glutathionylation in LHON through antioxidant strategies.

## 4. Materials and Methods

### 4.1. Patients

This study was approved by the Ethical Committee (Comité de Protection des Personnes—CPP OUEST II Angers, France; Date of approval: November, 23, 2015; Project identification: Collection Biologique de Maladies neurogénétiques; codes: DC-2011-1467 et AC-2014-2329) of the University Hospital of Angers (Comité de Protection des Personnes CPP Ouest II—Angers, France; Identification number: CPP CB 2014/02; Declaration number: DC−2011–1467 and Authorization number: AC−2012–1507), adhering to the tenets of the Declaration of Helsinki for human research. Written informed consent was obtained from all individuals participating in the study. Primary skin fibroblasts from eleven individuals, each carrying one pathogenic variant of mtDNA associated with LHON (m.11778G>A, m.3460G>A, and m.14484T>C, according to the mtDNA reference sequence rCRS/ NC_012920.1), and from seven healthy subjects, hereafter referred to as controls, were included in the study. All patients with LHON had typical clinical manifestations of the disease, which was genetically confirmed. The healthy control subjects were free of any ophthalmic condition and more generally, any mitochondrial disease.

### 4.2. Cell Cultures

Fibroblasts were cultured in a medium consisting of two-thirds Dulbecco’s modified eagle medium (DMEM-F12, Pan Biotech, Aidenbach, Germany), supplemented with 10% foetal bovine serum (Pan Biotech) at 37 °C, 5% CO_2_. To avoid artefacts due to senescence, all the experiments were conducted on fibroblast cultures with fewer than 25 passages [33].

### 4.3. Complex I Enzymatic Activity and Inhibition

Complex I activity was measured using cell lysates. Cells were disrupted by freezing in liquid nitrogen followed by rapid thawing at 37 °C. Lysates were then enriched in mitochondria by centrifugation (16,000× *g*, 1 min at 4 °C), washed once in cell buffer (50 mL/10^6^ cells), diluted at 1/5 and sonicated for 6 × 5 s on ice with a Branson Sonic Power sonicator (SmithKline Company, Brentford, London). NADH ubiquinone reductase (NUR) activity was measured as described previously [34] at 37 °C on a UVmc2 spectrophotometer (SAFAS). NADH (0.10 mM) was added to initiate the reaction. Rotenone (5 µM) was used to determine the background rate. Complex I activity was normalized with respect to citrate synthase activity that was assayed by a standard procedure [35].

To investigate the effect of experimental partial inhibition of Complex I on protein glutathionylation, healthy fibroblasts were treated for 48 h either by the vehicle (ethanol 1/2500) or 1 μM of rotenone, before measuring Complex I enzymatic activities.

### 4.4. Mitochondrial ROS Production

ROS production was measured simultaneously with oxygen consumption at 37 °C and atmospheric pressure in respiratory buffer using an O2k-Fluorometer (Oroboros Instrument, Innsbruck, Austria) equipped with a two-channel fluorescence optical setup to monitor the oxygen level and fluorescence. H_2_O_2_ production was monitored using 10 µM of the H_2_O_2_-sensitive probe Amplex^®^ Red (Molecular Probes, Eugene, Oregon, USA) (excitation 525 nm/emission filter 580 nm). One U/mL horse radish peroxidase and 5 U/mL superoxide dismutase were added to the chamber to convert superoxide into H_2_O_2_. Calibrations were carefully performed before, during, and at the end of each experiment with stepwise additions of 0.1 µM H_2_O_2_. Dedicated CI and CII-linked ROS production was sequentially analyzed in each sample using substrates of CI and CII as follows: first, NADH supply to CI was induced by adding 2.5 mM pyruvate and 5 mM malate (+ADP, 1.5 mM). Then, NADH supply was further increased by the addition of 5 mM glutamate. Finally, succinate (10 mM) and rotenone (5 µM) were added to measure the ROS production through CII electron flow. Specific H_2_O_2_ fluxes were calculated in real-time using DatLab software (OROBOROS Instruments, Innsbruck, Austria) from the positive time derivative of the resorufin signal over time. Six LHON and six control fibroblasts were explored in duplicate.

### 4.5. Measurements of Phosphorylating Respiration and ATP Production in LHON-Permeabilized Fibroblasts

The maximal phosphorylating respiration rate and corresponding mitochondrial ATP synthesis were determined in LHON (*n* = 7, 1 m.3460G>A, 2 m.14484T>C, and 4 m.11778G>A) and control (*n* = 7) fibroblasts permeabilized by digitonin exposure, as described previously [36]. Cells were resuspended in a respiratory buffer (10 mM KH_2_PO_4_, 300 mM mannitol, 10 mM KCl, and 5 mM MgCl_2_, pH = 7.4) supplemented with 2 mM iodoacetate and 2 mM EDTA, so as to prevent glycolytic ATP synthesis and ATP hydrolysis by cellular ATPases. The respiratory rates of 3 to 5 × 10^6^ cells were recorded at 37 °C in 2 mL glass chambers using a two-channel, high-resolution Oxygraph respirometer (Oroboros, Innsbruck, Austria). ATP synthesis was started either by addition of 5 mM malate and 2.5 mM pyruvate (Complex I-linked substrates), or of 5 mM malate, 2.5 mM pyruvate, and 10 mM succinate (complexes I+II substrates), and phosphorylating respiration was induced by the subsequent addition of 1.5 mM ADP. Four aliquots were sampled each minute, quenched with an equal volume of 1% trichloroacetic acid and TCA solution and neutralized by adding 25 mM 4-(2-hydroxyethyl)-1-piperazineethanesulfonic acid (HEPES), 2 mM EDTA, pH = 7.75 buffer. The ATP synthesized in situ was measured using the Enliten ATP assay (Promega, Madison, WI). Luminescence was measured on a Miniluma luminometer (Berthold Technologic, Bad Wilbad, Germany) using a 10-s integration period. Standardization was performed with known quantities of ATP measured under the same conditions. Protein concentrations within the chambers were determined by standard BCA assay.

### 4.6. Enrichment of S-glutathionylated Proteins

Culture media were removed from each well, and cells were rinsed three times with ice-cold PBS. One ml of ice-cold 10% (*w/v*) TCA was added to each well and kept on ice for 10 min. Cells were then scraped, and the suspension was transferred into a 1.5 mL microtube. All microtubes were kept on ice for 20 min and vortexed every 10 min. Samples were stored at −80 °C until further processing.

The overall workflow of the proteome-wide analysis of *S*-glutathionylation is given in Figure 4. Cell lysates were centrifuged at 14,000× *g* for 30 min at 4 °C. Denaturing buffer containing 6 M urea, 200 mM Tris-HCI, pH = 8.5, 10 mM EDTA, and 4% (*w*/*v*) SDS was freshly prepared before use. The protein pellet was rinsed once with 0.5 mL ice-cold 10% (*w/v*) TCA, followed by a second rinse with 0.2 mL ice-cold 5% (*w/v*) TCA. The pellet was then re-dissolved in 80 µL denaturing buffer, and the total protein concentration was determined using the BCA assay. An aliquot of 100 µg of total protein was used for alkylation with light ICAT reagent for 2 h. Proteins were precipitated using 0.5 mL of −20 °C cold acetone and maintained at −20 °C overnight. The acetone precipitate was centrifuged at 14,000× *g* for 30 min at 4 °C, and the protein pellet was rinsed twice with 0.5 mL −20 °C cold acetone to remove excess light ICAT reagent. The protein pellet was resolubilized in denaturing buffer and prepared for deglutathionylation as follows: the protein solution was loaded onto a Nanosep^®^ centrifugal device with Omega™ Membrane 30K (Pall Life Sciences, Ann Arbor, MI, USA), and SDS was removed by washing three times with 6 M urea, followed by three washes with 50 mM ammonium bicarbonate to condition the membrane for the subsequent deglutathionylation step. For deglutathionylation, the following mixture was added: 10 mM GSH, 10 mM NADPH, 4 U/mL GR, and 0.5 U/mL Grx1 in 100 µL 50 mM ammonium bicarbonate and incubated for 1 h at 37 °C on the spin column. Excess GSH was removed by washing four times with 50 mM ammonium bicarbonate. Newly reduced cysteines were labelled with heavy ICAT reagent for two hours, followed by removal of excess heavy ICAT by washing three times with 50 mM ammonium bicarbonate. Proteins were then digested with trypsin (1:30 trypsin/protein ratio) for 12–16 h at 37 °C. Peptides were eluted with 50 mM ammonium bicarbonate and 0.5 M sodium chloride. Peptides were purified using avidin affinity chromatography according to the manufacturer’s protocol (ABSciex, Framingham, MA, USA). Briefly, an Affinity column was cleaned with Affinity Elute buffer, followed by equilibration with Affinity Load buffer. Samples were further diluted with Affinity Load buffer and loaded onto the Affinity column. The column was washed with Affinity Load buffer, followed by Affinity Wash 1, Affinity Wash 2, and lastly with distilled water. The peptides were eluted with Affinity Elute buffer and were dried without heating. The Affinity (Biotin) tag was cleaved with 95:5 mixtures of Cleavage reagent A (trifluroacetic acid) and Cleavage reagent B. The cleavage reaction was incubated at 37 °C for 2 h and dried without heating using speedvac. Finally, the biotin tag was cleaved, and samples were desalted using UltraMicro Spin Columns (Nest Group, Southborough, MA, USA) before LC/MS/MS analysis.

### 4.7. LC-MS/MS Analysis

An Ultimate 3000 nanoLC system (Dionex, Thermo Fisher Scientific, MA, USA) coupled to an ABSciex 5600 TripleTOF (ABSciex, Framingham, MA, USA) was used for proteomic analysis. Peptide samples were separated on a 50 cm × 75 µm i.d. Acclaim PepMap RSLC C18 column (Dionex, Thermo Fisher Scientific, MA, USA), which was connected to a spray tip (New Objectives, Woburn, MA). Peptides samples were loaded onto a trap column (Acclaim PepMap 100 C18, 2 cm × 75 µm i.d., Dionex, Thermo Fisher Scientific, MA, USA) at a flow rate of 5 µL/min. After a 3-min wash with loading buffer (2:98 *v/v* of ACN/water with 0.1% formic acid), the system was switched into line with the C18 analytical capillary column. The mobile phases were as follows: Solvent A: ACN/water with 0.1% formic acid; 2:98 *v/v*; Solvent B: 2/98 *v/v* of water/ACN with 0.1% formic acid. A step linear gradient of Solvent B (2:98 *v/v* of water/ACN with 0.1% formic acid) from 5% to 25% for 10 min, 25–60% for 9 min, and lastly, 60–95% for 1 min at a flow rate of 300 nL/min was utilized for this analysis. A third-generation Nanospray Source was used, and MS settings with Analyst TF 1.6 software (ABSciex, Framingham, MA, USA) were as follows: ionspray voltage floating, 2200 V; curtain gas, 30 psi; ion source gas 1, 12 psi; interface heater temperature, 125 °C; declustering potential, 100 V. Data were acquired using TOF MS under Hi Sensitivity product ion mode. TOF-MS scan parameters were set as follows: 0.25 s TOF MS accumulation time in the mass range of 350 to 1250 Da was followed by MS/MS scanning based on the following parameters: mass range was set at 100 to 1500 Da; switching criteria were set to ions greater than m/z = 350 and smaller than m/z = 1250 with a charge state of 2 to 5 and an abundance threshold greater than 120 cps. Former target ions were excluded for 4 s and also excluded after one repeat. The maximum number of candidate ions to monitor per cycle was 20 spectra with an accumulation time of 100 ms.

### 4.8. Data Analysis

The raw MS data were processed using ProteinPilot software 4.5 (ABSciex, Framingham, MA, USA) with a database search using Uniprot (Sept 2010 release, 40516 proteins searched). Searching parameters were set as follows: (1) sample type: cleavable ICAT; (2) Cys Alkylation: none; (3) digestion: trypsin; (4) instrument: TripleTOF 5600; (5) species: Homo sapiens; (6) ID focus: biological modifications. All peptides identified from the database were filtered according to the following criteria: at least 95% confidence and possessing a heavy-to-light (H:L) ratio of at least 0.01. Peptides that did not meet the criteria were removed from further analysis. For case/control experiments, the average H:L values across all LHON cases (*n* = 11 in duplicate) or controls (*n* = 7 in duplicate) were calculated, and then the [H:L disease / H:L control] value for each peptide was obtained. Similarly, in the Complex I inhibition treatment, the average H:L values across triplicate sets of peptide data from either vehicle-treated control (*n* = 6) or rotenone-treated cells (*n* = 6) were computed, and the ratios of [H:L treated / H:L control] for each peptide were then obtained. A particular peptide was considered to be significantly glutathionylated if the [H:L disease / H:L control] or [H:L treated / H:L control] ratio exceeded 1.5-fold [17] and was subjected to further pathway analysis described below.

Gene Ontology (GO) analysis was performed using the Database for Annotation, Visualization and Integrated Discovery (DAVID, v6.8) [37]. Ingenuity Pathway Analysis (IPA, Qiagen) was used to identify networks of interacting proteins. STRING (v.11) [38] was used to establish the protein–protein association network. Proteo-metabolomic mapping was performed using the “Joint pathway analysis” module in “Metaboanalyst 4.0” [19].

### 4.9. Simulations of the Molecular Dynamics (MD) of Native and Glutathionylated ANT2

MD simulations were performed to understand the effect of *S*-glutathionylation on the structure and dynamics of ANT2. As the crystal structure of human ANT2 is not yet resolved, we performed homology modeling based on the bovine mitochondrial ADP–ATP carrier (PDB ID: 2c3e). Supplemental Figure S3 shows the alignment of the human and the bovine mitochondrial ANT2 protein. There are four cysteine residues in the human ANT2 molecule, and Cys160 was glutathionylated based on the experimental results from proteomic analysis. The native and glutathionylated ANT2 molecules were then subjected to subsequent MD simulations. Both proteins were embedded into a membrane patch of 152 POPC lipid molecules. Protein molecules were modeled using the AMBER99sb force field, and the lipid molecules were described using the Slipid parameters, which are compatible with the AMBER force field [39]. The TIP3P water model was used to solvate the protein–membrane system, and counter ions were added to neutralize the system. First-energy minimization of 500 steps using a steep descent algorithm was performed, followed by 100 ps of molecular dynamics simulations with protein atom positions restrained. Then, production MD simulations were run for 500 ns for both native and glutathionylated ANT2. During the molecular dynamics simulations, the short-range non-bonded interactions (electrostatic and Van der Waals potentials) were treated using a cut-off value of 1.0 nm, while the long-range electrostatic interactions were calculated using PME [40]. The simulations were carried out in NPT ensemble in which the temperature and pressure were maintained to 300 K and 1 bar, respectively. The simulations were performed using the GROMACS package [41].

### 4.10. Statistical Analysis

For proteomic analysis and quantitative analyzes of Complex I activities, differences between groups were evaluated by the Mann–Whitney statistical test. Results were considered significant when the *p* < 0.05.

## Figures and Tables

**Table 1:** Description of the cohort of patients and controls.

Subjects	Gender	Age (Years)	Passages	LHON Mutations (Rate)
Patient 1	M	20	15	m.11778G>A 97%
Patient 2	F	44	15	m.11778G>A 100%
Patient 3	M	26	13	m.11778G>A 100%
Patient 4	M	39	17	m.11778G>A 100%
Patient 5	F	58	8	m.11778G>A 97%
Patient 6	M	23	10	m.11778G>A 97%
Patient 7	M	22	17	m.14484T>C 100%
Patient 8	M	19	10	m.14484T>C 100%
Patient 9	M	38	12	m.3460G>A 100%
Patient 10	F	42	11	m.11778G>A 100%
Patient 11	M	22	12	m.11778G>A 96%
Control 1	M	8	21	
Control 2	F	24	12	
Control 3	F	37	9	
Control 4	M	30	11	
Control 5	F	28	9	
Control 6	F	56	20	
Control 7	M	30	19	

## Supplementary Materials

Supplementary materials can be found at https://www.mdpi.com/1422-0067/21/8/3027/s1.

## Abbreviations

AIF	Apoptosis inducing factor
ANT2	Adenine nucleotide translocase 2
ATP	Adenosine triphosphate
DNA	Deoxyribonucleic acid
GPx	Glutathione peroxidase
Grx1	Glutaredoxin 1
GSH	Glutathione
GSSG	Glutathione disulfide
ICAT	Isotope-coded affinity tag
LHON	Leber’s Hereditary Optic Neuropathy
MPTP	Mitochondrial permeability transition pore
mtDNA	Mitochondrial DNA
ND	NADH dehydrogenase
PPARα	Peroxisome proliferator-activated receptor α
PTMs	Post-translational modifications
RMSD	Root mean square deviation
UCP	Uncoupling protein
VDAC1	Voltage-dependent anion-selective channel protein 1
