# Supplementary figures and images for: Increased Protein S-Glutathionylation in Leber’s Hereditary Optic Neuropathy (LHON)
Source: Int J Mol Sci. 2020 Apr 24;21(8):3027. doi: 10.3390/ijms21083027 (PMC7215361; doi:10.3390/ijms21083027)

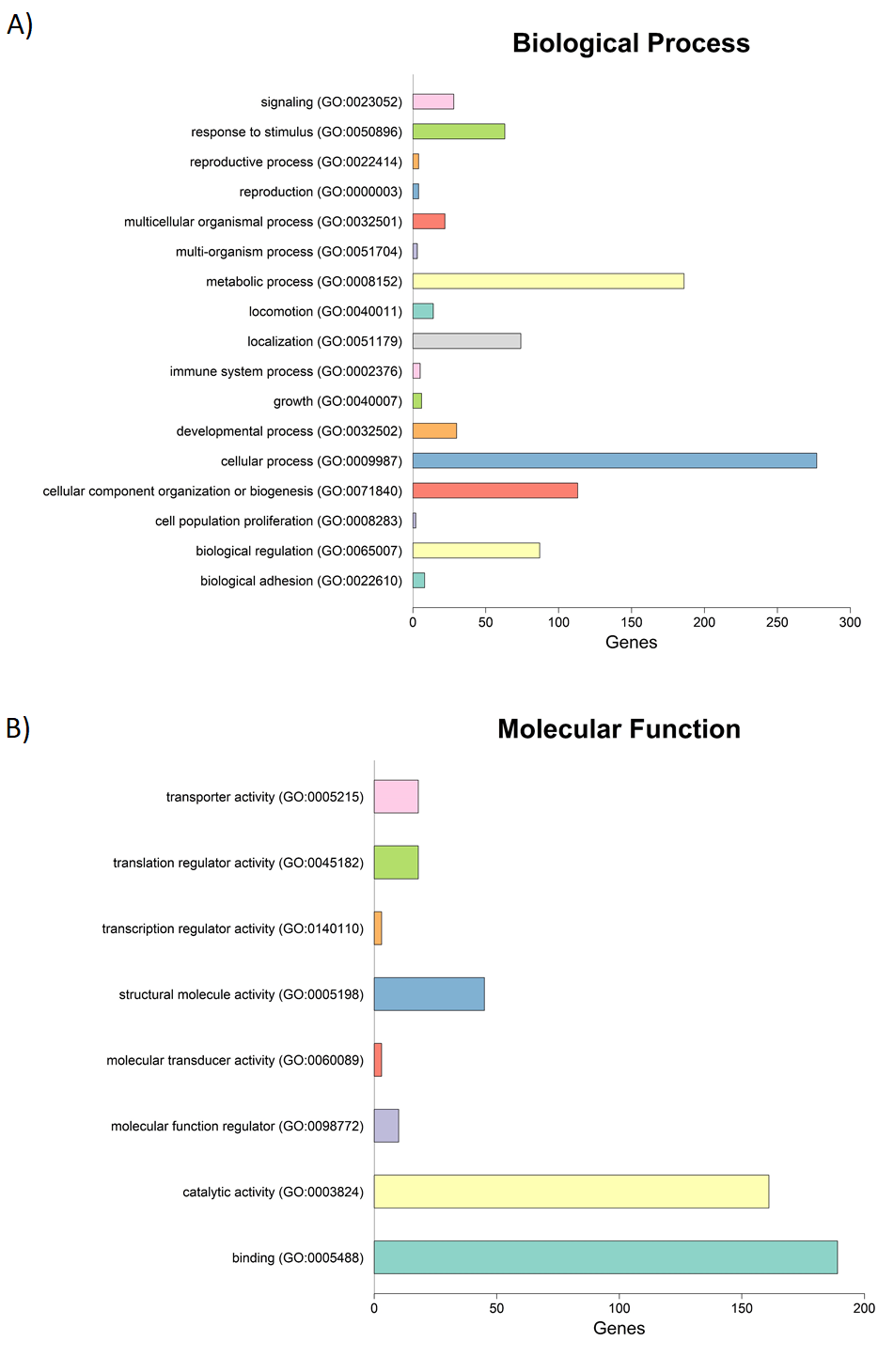

Supplement: Supplementary file 1 [file ijms-21-03027-s001.zip › Supplementary Figures/Supplementary Figure 1.tif]

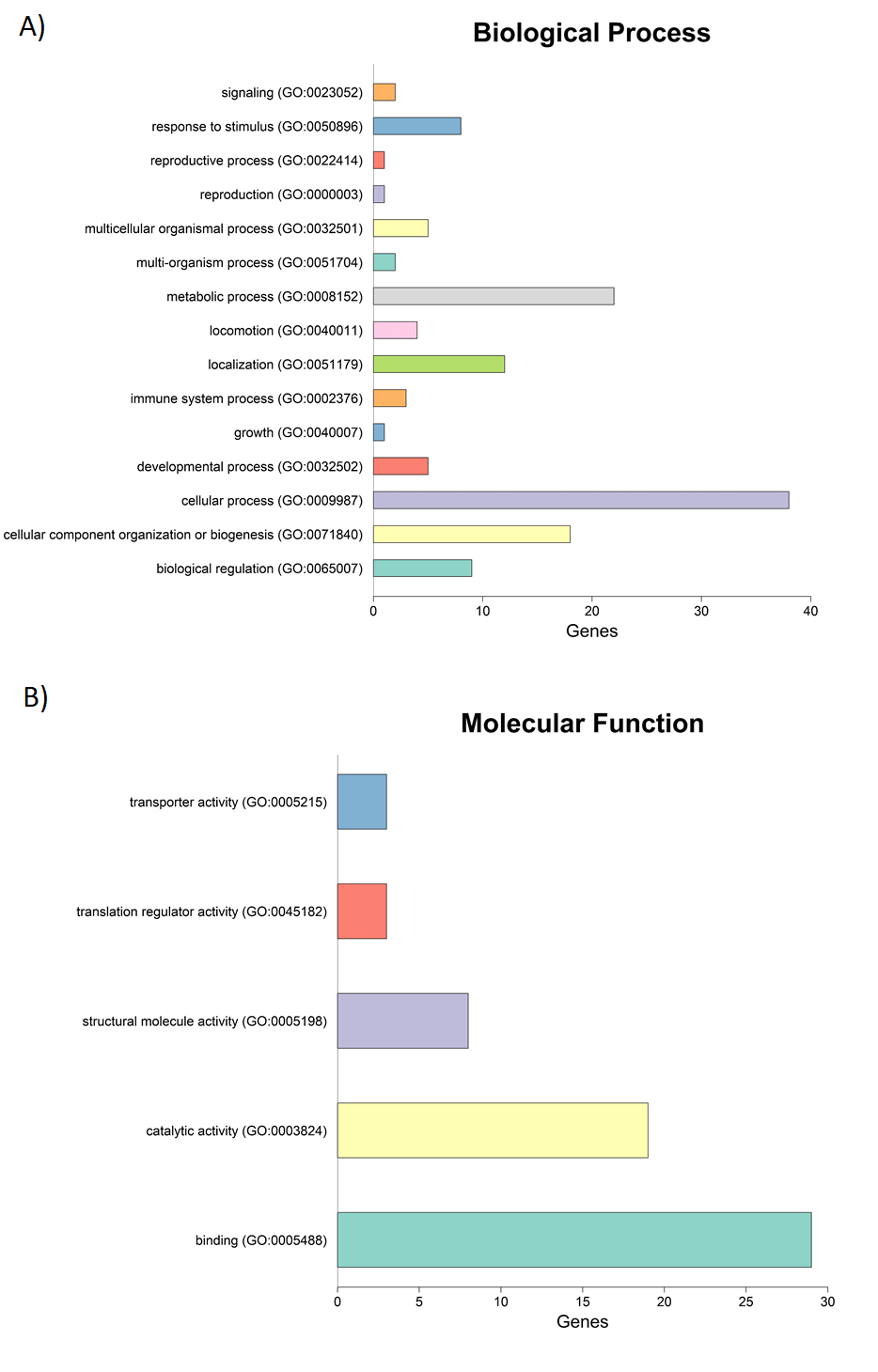

Supplement: Supplementary file 1 [file ijms-21-03027-s001.zip › Supplementary Figures/Supplementary Figure 2.tif]

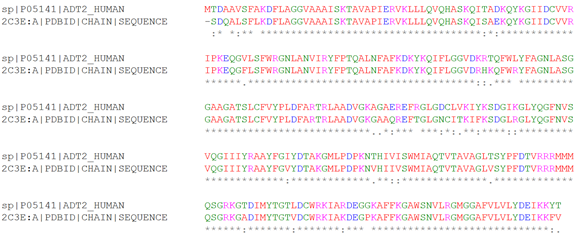

Supplement: Supplementary file 1 [file ijms-21-03027-s001.zip › Supplementary Figures/Supplementary Figure 3.tif]
